# *Caenorhabditis elegans* transposable elements harbor diverse transcription factor DNA-binding sites

**DOI:** 10.1093/g3journal/jkac009

**Published:** 2022-01-17

**Authors:** Jacob M Garrigues, Amy E Pasquinelli

**Affiliations:** Section of Molecular Biology, Division of Biology, University of California, San Diego, CA 92093, USA

**Keywords:** transposons, gene expression, genome evolution, *Caenorhabditis elegans*

## Abstract

Transposable elements are powerful agents of evolution that can diversify transcriptional programs by distributing transcription factor DNA-binding sites throughout genomes. To investigate the extent that transposable elements provide transcription factor-binding motifs in *Caenorhabditis elegans*, we determined the genomic positions of DNA-binding motifs for 201 different transcription factors. Surprisingly, we found that almost all examined transcription factors have binding motifs that reside within transposable elements, and all types of transposable elements have at least 1 instance of a transcription factor motif, demonstrating that transposable elements provide previously unappreciated numbers of transcription factor-binding motifs to the *C. elegans* genome. After determining the occurrence of transcription factor motifs in transposable elements relative to the rest of the genome, we identified DNA-binding motifs for 45 different transcription factors that are greater than 20-fold enriched within transposable elements compared to what would be expected by chance. Consistent with potential functional roles for these transposable element-enriched transcription factor-binding sequences, we determined that all transcription factor motif types found in transposable elements have instances of residing within accessible chromatin sites associated with transcription factor binding. The overwhelming majority of transcription factor-binding motifs located within transposable elements associate with their cognate transcription factors, suggesting extensive binding of transcription factors to sequences within transposable elements. In addition, transposable elements with accessible or transcription factor-bound motifs reside in the putative promoter regions of approximately 12% of all protein-coding genes, providing widespread possibilities for influencing gene expression. This work represents the first comprehensive analysis of transposable element–transcription factor interactions in *C. elegans* and demonstrates that transposable element-provided transcription factor-binding sites are prevalent in this important model organism.

## Introduction

Substantial portions of most animal and plant genomes are composed of repetitive sequences, many of which are derived from transposable elements (TEs) (Wells and Feschotte 2020). While initially thought to be functionally dormant and often described as “junk DNA” in the past, more recent work has shown that TEs can serve various functions, such as influencing gene expression by providing regulatory elements in *cis* (Sundaram and Wysocka 2020). TE-derived *cis*-regulatory elements can affect gene expression in diverse manners, such as by supplying boundary elements that contribute to the formation of topologically associated domains (TADs), silencer elements able to promote the formation of repressive chromatin that spreads into and silences neighboring genes, and enhancer as well as promoter elements containing binding sites for transcription factors (TFs) that influence the expression of distally and proximally located genes, respectively (Sundaram and Wysocka 2020). Highlighting the potential of TEs to act as promoter elements, an early analysis of the human genome estimated that 25% of promoter regions have sequences derived from TEs (Jordan *et al.* 2003), and a comprehensive analysis of 26 orthologous pairs of TFs between humans and mice determined that 20% of TF-binding sites reside within TEs, with some TFs having 40% of their binding sites provided by TEs (Sundaram *et al.* 2014).

The mobilization of TEs provides the ability to rapidly diversify existing gene expression programs by distributing functional *cis*-acting elements throughout host genomes. Intact functional elements can be inserted into new regions during a single transposition event, circumventing an extended process involving multiple single-nucleotide changes (Sundaram and Wang 2018). Alternatively, TEs can provide partial sequences that evolve into functional elements over time (Sundaram and Wysocka 2020). While most TE insertions are predicted to be deleterious or selectively neutral in nature, some insertion events can be beneficial and subsequently co-opted and maintained by their hosts (Chuong *et al.* 2017). Examples of regulatory networks that contain co-opted TEs include the mammalian interferon response (Chuong *et al.* 2016), early mouse development (Todd *et al.* 2019), and stem cell pluripotency in human embryos (Fuentes *et al.* 2018; Pontis *et al.* 2019).

Previously, we showed that Helitron TEs in the model organism *Caenorhabditiselegans* provide substantial numbers of HSF-1-binding sites that have incorporated new genes into the heat-shock response (HSR) (Garrigues *et al.* 2019). In this study, we set out to estimate the potential that TE-derived sequences have to affect gene expression in *C. elegans*. We show that TEs make up substantial portions of protein-coding gene promoters, as well as introns and distal intergenic regions. After further examination, we found that TEs are enriched for diverse TF DNA-binding motifs, and TE-supplied TF motifs make up notable amounts of the total numbers of motifs found in the genome. Many TE-derived TF motifs reside within open chromatin sites associated with TF binding and/or are directly bound by their cognate TFs. In a specific example, we find that genes either containing or lacking TE-derived promoter LSL-1 motifs reside in chromatin environments consistent with gene expression but largely display differences in their enriched gene ontology (GO) terms. Finally, orthologous genes shared between *C. elegans* and *Caenorhabditisbriggsae* contain similar TF-motif-containing TEs, some of which may represent the conservation of ancient TE insertions. As the first comprehensive analysis of TE-TF interactions in *C. elegans*, our studies reveal that TEs provide numerous TF-binding sites with widespread potential for influencing gene expression in this important model organism.

## Materials and methods

### TE, genomic feature, and gene annotations

*Caenorhabditis* *elegans* TEs were obtained from the RepeatMasker track (Smit *et al.* 2013) using Repbase TE annotations (Jurka 2000) available from the UCSC Genome Browser (genome build ce11) (Kent *et al.* 2002) and by filtering for the following repeat class terms: “DNA,” “DNA?,” “LINE,” “LTR,” “RC,” and “SINE.” *Caenorhabditisbriggsae* TEs were obtained from WormBase (version WS280) (Harris *et al.* 2020). Positions of *C. elegans* protein-coding genes and one-to-one orthologs shared between *C. elegans* and *C. briggsae* were obtained from ParaSite (version WBPS15) (Howe *et al.* 2016, 2017), while *C. elegans* operons were obtained from WormBase (version WS280). Coordinates for coding exons, 5’UTRs, 3’UTRs, and introns were acquired from the UCSC Genome Browser using WormBase annotations (version WS245). Promoter regions are defined as the space within 2.5 kb upstream of protein-coding genes or operons. The overlap of TEs with genomic features was determined using the intersect function of BEDTools v2.29.2 (Quinlan and Hall 2010).

### TF DNA-binding motif collection and genome scanning

*Caenorhabditis* *elegans* TF motifs with direct experimental evidence for their preferred binding sequences were obtained from the Cis-BP database v2.00 (Weirauch *et al.* 2014). To convert Cis-BP motifs into a format suitable for genome scanning using FIMO (Grant *et al.* 2011), the chen2meme utility that is part of the MEME suite v4.11.2 (Bailey *et al.* 2009) was used with the following piped commands: sed ‘1d’ | cut -f2- | chen2meme. After conversion, FIMO v4.11.2 was used to scan and identify motif positions in the *C. elegans* (ce11) and *C. briggsae* (cb4) genomes with a statistical cutoff of *P* < 1e−04 using the following command: fimo –max-stored-scores 100000000 –max-strand. To account for biases in the genomic distribution of individual bases, a zero-order Markov background model was generated using the fasta-get-markov utility included with the MEME suite and used during motif scanning. To determine whether scanned TF motifs reside within TE sequences, the intersect function of BEDTools (Quinlan and Hall 2010) was then used to identify TF motifs that completely reside within annotated TE positions.

### ATAC-seq data collection and processing

ATAC-seq data used in this study were previously published (Daugherty *et al.* 2017). The first read from each mate pair was aligned to the reference genome (ce11) using Bowtie2 v2.4.2 (Langmead and Salzberg 2012) in local alignment mode with the following parameters: –very-sensitive-local. By using local alignment mode, adapter sequences and low-quality base calls were soft clipped from reads. In addition, multimapping reads were assigned to single positions at random with these settings. Peak summits were then called (*q* < 0.05) and bedGraphs of SPMR-normalized signal were generated individually for each available developmental stage (early embryo, L3, and young adult) using combined biological replicates of experimental data with MACS2 v2.2.7.1 (Zhang *et al.* 2008) and the following ATAC-seq-specific settings: macs2 callpeak –nolambda –nomodel –extsize 150 –shift -75 -g ce –keep-dup auto -B –SPMR -q 0.05 –call-summits. By using the–keep-dup auto parameter, the number of duplicate reads allowed depends on the total number of reads present. Peak summits were extended 50 bp in both directions to yield 101-bp summit regions, and summit regions from all developmental stages were subsequently merged using BEDTools (Quinlan and Hall 2010).

### ChIP-seq data collection and processing

TF ChIP-seq data were obtained from the modERN database (Kudron *et al.* 2018), except for HSF-1 (Li *et al.* 2016). Single-ended reads were aligned to the genome (ce11) using Bowtie2 (Langmead and Salzberg 2012) in local alignment mode with the following parameters: –very-sensitive-local. By using local alignment mode, adapter sequences and low-quality base calls were soft clipped from reads. In addition, multimapping reads were assigned to single positions at random with these settings. Peak summits were then called (*q* < 0.01) and bedGraphs of SPMR-normalized signal were generated individually for each available developmental stage using combined biological replicates of experimental and control data with MACS2 (Zhang *et al.* 2008) and the following settings: macs2 callpeak –nomodel -g ce –keep-dup auto -B –SPMR -q 0.01 –call-summits –scale-to large. By using the –keep-dup auto parameter, the number of duplicate reads allowed depends on the total number of reads present. Peak summits were extended 50 bp in both directions to yield 101-bp summit regions, and summit regions from all developmental stages for each individual TF were then merged using BEDTools (Quinlan and Hall 2010). To match LSL-1 ChIP-seq data obtained from young-adult-staged worms, young-adult histone modification ChIP-seq data (H3K4me3, H3K27me3, and H3K36me3) were obtained from Jänes *et al.* (2018), while young-adult Pol II data were obtained from the modMine database (release 33) for modENCODE data (Contrino *et al.* 2012). For signal visualization, bedGraphs of SPMR-normalized histone modification and Pol II ChIP-seq data from combined biological replicates were generated using MACS2 with the following settings: macs2 callpeak –nomodel -g ce –keep-dup auto -B –SPMR.

### Heatmaps

Heatmaps depicting TF-binding motifs in TEs were generated using R v4.0.3 (R Core Team 2020) combined with the heatmap.2 function found in the gplots v3.1.1 package (Warnes *et al.* 2020). Colors were generated using the RColorBrewer v1.1-2 package and the included YlOrRd sequential palette (Neuwirth 2014). Data were organized on the *x*-axes by implementing Ward.D hierarchical clustering following the calculation of Euclidean distances using the hclust and dist functions of R, respectively. Heatmaps of ChIP-seq and ATAC-seq signal surrounding LSL-1 peak summits in promoter regions were generated using the computeMatrix and plotHeatmap tools part of the Deeptools2 suite (Ramírez *et al.* 2016) to process bedGraphs of SPMR-normalized data produced by MACS2 (Zhang *et al.* 2008).

### GO term enrichment analyses

Protein-coding genes with TE-residing TF motifs in their promoter regions were identified using the BEDTools intersect function (Quinlan and Hall 2010). LSL-1-bound genes were then analyzed with the Gene Set Enrichment Analysis webtool found at WormBase (version WS282) (Angeles-Albores *et al.* 2018) to find any significantly enriched GO terms using a *q*-value cutoff of 0.05. To reduce redundancy, resulting GO terms were compared to the whole UniProt database and filtered with REViGO (Supek *et al.* 2011) using the SimRel semantic similarity measure and an allowed similarity of 0.5.

## Results

### Transposable elements in *C. elegans* make up substantial portions of protein-coding gene promoters

**Fig. 1.**
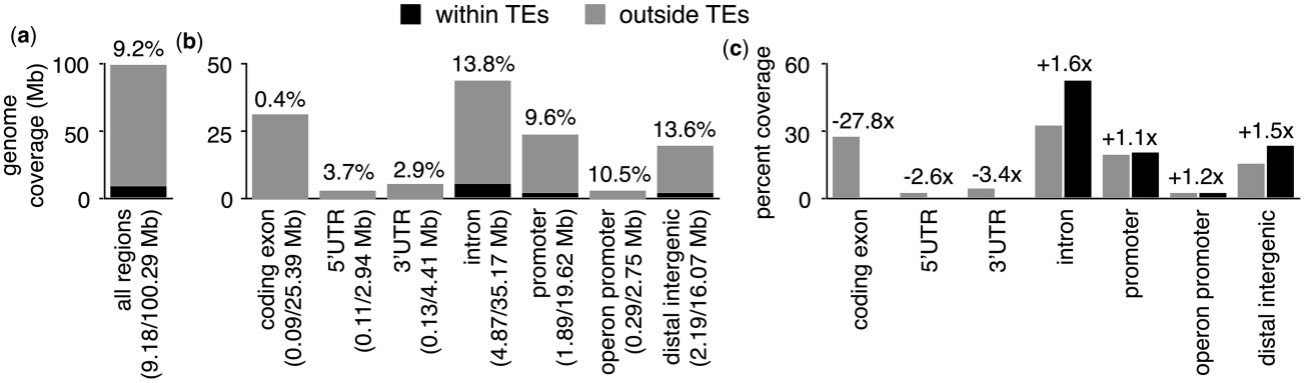
Distribution of TEs in *C. elegans* genomic regions. a) Bar chart depicting the proportion of the *C. elegans* nuclear genome annotated as TE (black) or not (gray). b) Bar chart showing the proportions of the genome covered by coding exons, UTRs, introns, promoters, operon promoters, or distal intergenic regions annotated as TE (black) or not (gray). Promoter regions are defined as regions within 2.5 kb upstream of protein-coding genes or operons, and for this analysis, overlapping coding exons, UTRs, and introns have been excluded from promoters. Due to overlapping exons, UTRs, and introns, the total amount of genomic space covered is greater than 100% of the genome. Distal intergenic regions are defined as the genomic space that lies outside of any other category. The percentage of each individual category that TEs comprise is displayed above each category. c) Bar chart depicting the percentages of the genome covered by coding exons, UTRs, introns, promoter regions, or distal intergenic regions that reside within (black) or outside of TEs (gray). Due to overlapping exons, UTRs, and introns, the total percentage of the genome covered by all categories is greater than 100%. Fold enrichments for regions within TEs compared to regions outside of TEs are shown above each genomic category.

Regions derived from TEs make up a substantial portion of protein-coding gene promoters in humans (Jordan *et al.* 2003), where they can provide sequences that affect gene expression in *cis* (Sundaram and Wysocka 2020). Previously, it was reported that TE insertions in *C. elegans* predominantly occur outside of information-rich exons and accumulate within introns, distal intergenic regions, and promoters (Laricchia *et al.* 2017). To determine if TEs make up a sizeable portion of the genomic space occupied by protein-coding gene promoters, we determined the proportion of the genome covered by all annotated TEs (Fig. 1a), as well as the proportions of protein-coding gene and operon promoters (Fig. 1b). Most TEs in *C. elegans* are thought to be inactive (Bessereau 2006), and the majority of annotated TEs present in the genome are TE remnants consisting of partial sequences (Storer *et al.* 2021). For comparative purposes, we also determined the proportions of coding exons, 5’UTRs, 3’UTRs, introns, and distal intergenic regions covered by TEs (Fig. 1b). Through this analysis, we determined that 9.2% of the *C. elegans* nuclear genome (9.18/100.29 Mb) is covered by TEs and their remnants (Fig. 1a). This differs from 12% to 16% previously reported (*C. elegans* Sequencing Consortium 1998; Sijen and Plasterk 2003; Laricchia *et al.* 2017), possibly due to differences between reference genome assemblies or annotated TEs. Interestingly, we found that 9.6% of the genomic space occupied by promoters (1.89/19.62 Mb) and 10.5% of operon promoters (0.29/2.75 Mb) are covered by TEs (Fig. 1b). Promoter regions are defined as regions within 2.5 kb upstream of protein-coding genes or operons with overlapping coding exons, UTRs, and introns removed. After considering exons, we determined that 0.4% of the space occupied by coding exons (0.09/25.39 Mb), 3.7% of 5’UTRs (0.11/2.94 Mb), and 2.9% of 3’UTRs (0.13/4.41 Mb) are covered by TEs (Fig. 1b). We also found that 13.8% of introns (4.87/35.17 Mb) and 13.6% of distal intergenic regions (2.19/16.07 Mb) are covered by TEs (Fig. 1b). From these observations, we conclude that TEs make up a notable portion of the genomic space occupied by promoters in *C. elegans*. Strikingly, we find that TE-derived sequences are within 2.5 kb upstream of 47.8% (9,561/19,987) of all protein-coding genes, raising the possibility that gene expression is affected by TE-provided sequences in a widespread manner.

To determine whether the observed coverage of promoters by TEs represents a relative enrichment or depletion, we compared the proportion of TEs to the proportion of the genome without TEs occupied by promoters (Fig. 1c). We also performed the same analyses for coding exons, UTRs, introns, and distal intergenic regions (Fig. 1c). We found that the coverage of promoters by TEs represents a 1.1-fold enrichment (26.3% of the non-TE genome compared to 29.2% of TEs), while the coverage of operon promoters represents a 1.2-fold enrichment (2.7% of non-TE regions compared to 3.2% of TEs) (Fig. 1c). Consistent with previously published results (*C. elegans* Sequencing Consortium 1998; Laricchia *et al.* 2017), the portion of coding exons covered by TEs represents a 27.8-fold depletion (27.8% of non-TE regions compared to 1.0% of TEs), while the coverage of 5’UTRs and 3’UTRs by TEs represents a 2.6-fold depletion (3.1% of non-TE regions compared to 1.2% of TEs) and 3.4-fold depletion (4.7% of non-TE regions compared to 1.4% of TEs), respectively (Fig. 1c). The portion of introns covered by TEs represents a 1.6-fold enrichment (33.3% of non-TE regions compared to 53.1% of TEs), and the coverage of distal intergenic regions by TEs a 1.8-fold enrichment (8.4% of non-TE regions compared to 15.3% of TEs). Taken together, our analyses reveal that the portion of promoters covered by TEs in *C. elegans* represents neither an enrichment nor depletion, suggesting they are more amenable to TE insertions or preservation compared to information-rich exons but less so than introns and distal intergenic regions.

### TEs harbor multiple types of TF DNA-binding motifs

**Fig. 2.**
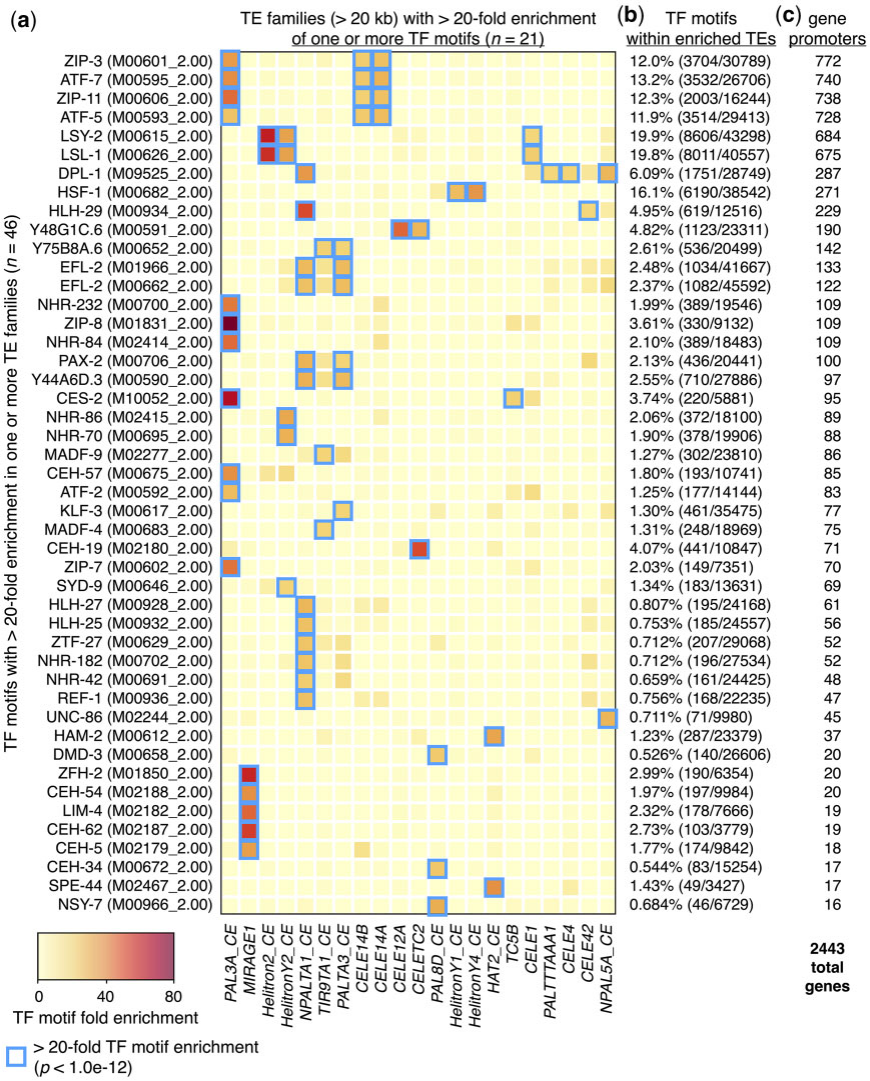
TEs enriched for TF-binding motifs reside within gene promoters. a) Heatmap depicting the fold enrichments of 46 TF-binding motifs (representing 45 different TF proteins) that are greater than 20-fold enriched in at least 1 type of TE that covers at least 20 kb of genomic space. Blue boxes indicate TE types that are greater than 20-fold enriched for an individual TF-binding motif (*P* < 1.0e−12, binomial test). Cis-BP database (Weirauch *et al.* 2014) identifiers for each TF-binding motif are shown in parentheses. Fold enrichment is defined as the proportion of each TF motif found in each TE type divided by the proportion of genomic space covered by each TE type. Heatmap columns are clustered by similarity, and rows are sorted by the numbers of genes identified in panel c. b) Percentage of indicated TF motifs that reside within repeats displaying greater than 20-fold enrichment from panel a (blue boxes), calculated as the total number of a given motif found within the TEs compared to the total number in the entire genome. c) Numbers of protein-coding genes that have TE-enriched TF-binding motifs from panel a (blue boxes) within their promoter regions. The total number of genes with TE-enriched motifs in their promoters is 2,443.

We previously showed that Helitron TEs provide HSF-1 DNA-binding sites (HSEs) that influence the expression of adjacent genes during heat shock in *C. elegans* (Garrigues *et al.* 2019). To determine whether TE-enriched TF binding sites are limited to Helitrons and HSEs in *C. elegans* or may be more prevalent and involve other kinds of TEs and TF-binding motifs, we scanned the nuclear genome for 229 DNA-binding motifs with direct experimental binding evidence obtained from the Cis-BP database (Weirauch *et al.* 2014) representing 201 different TFs. After performing this analysis, we found that almost all (223/229, or 97.4%) scanned TF DNA-binding motifs occur at least once within TEs or their remnants (Supplementary Table 1). To identify TE-residing TF-binding motifs with greater potential for influencing gene expression, we focused our subsequent analyses on TEs that cover more than 20 kb of the genome and looked within those TEs for TF motifs that are greater than 20-fold enriched compared to what would be expected by chance (*p* < 1.0e−12, binomial test) (Fig. 2a). This yielded 46 different TF-binding motifs representing 45 TFs that are highly enriched in 21 different types of TEs (Fig. 2a). For many of these TF motifs, the numbers found within TEs represent significant portions of all observed genomic motifs (Fig. 2b). For example, almost 20% of all LSY-2 and LSL-1 motifs completely reside within *Helitron2_CE*, *HelitronY2_CE*, and *CELE1* TE sequences (Fig. 2b). Many highly enriched TF motifs are present in consensus TE sequences obtained from Dfam (version 3.4) (Storer *et al.* 2021) (Supplementary Table 2), suggesting they were already present during transposition rather than subsequently acquired through evolutionary forces.

To determine the extent that TE-enriched TF motifs reside within promoter regions, we compared the positions of these motifs to the promoter regions of protein-coding genes. For this and subsequent analyses, promoters are defined as regions within 2.5 kb upstream of genes and include overlapping genomic features. Through this analysis, we found that a total of 2,443 or approximately 12% of all protein-coding genes have TE-enriched TF motifs in their promoters (Fig. 2c, Supplementary Table 3). These findings demonstrate that TF motifs found in TEs are prevalent in *C. elegans*, and TEs contain substantial portions of genomic TF motifs. Furthermore, TE-enriched TF motifs reside in the putative promoter regions of numerous genes, where they potentially influence their expression.

### Substantial portions of accessible TF DNA-binding motifs are found within TEs

**Fig. 3.**
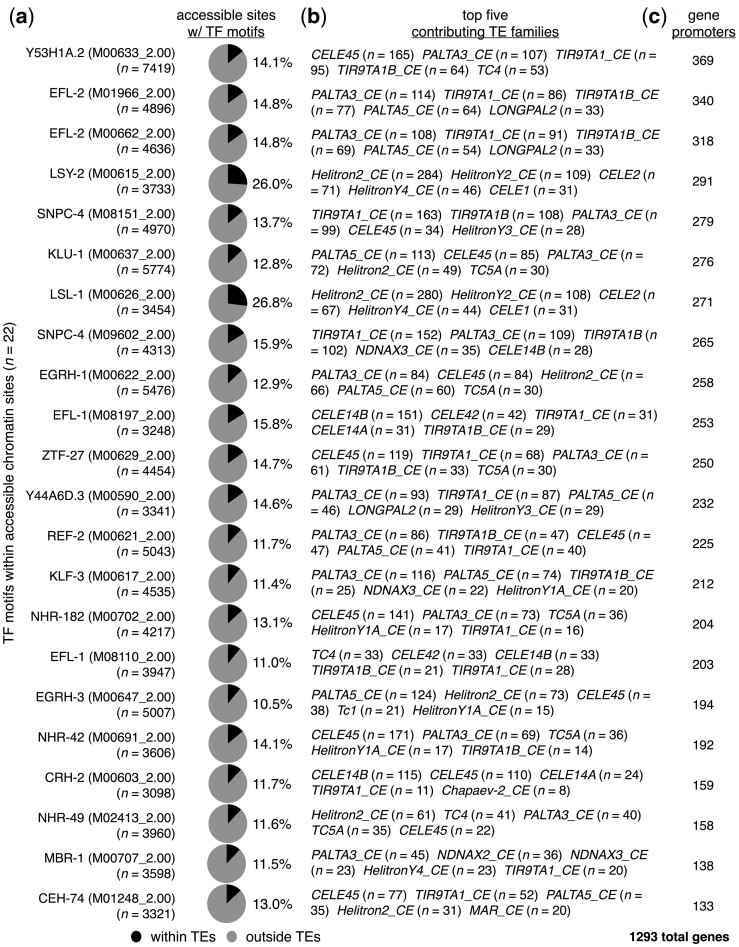
TF motifs within TEs reside within an open chromatin environment. a) Pie charts displaying the proportions of accessible chromatin sites containing the indicated TF-binding motifs that reside within (black) or outside of (gray) annotated TEs. Accessible sites were identified using ATAC-seq data from a previous publication (Daugherty *et al.* 2017). TF-binding motif identifiers from the Cis-BP database (Weirauch *et al.* 2014) are shown in parentheses. Only TF motifs with greater than 10% of accessible motifs within TEs are displayed. b) List of the top 5 TEs that have the greatest numbers of each indicated TF motif within accessible chromatin sites. c) Number of protein-coding genes that have TEs containing accessible TF motifs from panel (a) residing within their promoter regions. The total number of genes with accessible TE-provided TF motifs in their promoters is 1,293.

Diverse TF-binding sites have been observed to occur within mammalian TEs (Wang *et al.* 2007; Bourque *et al*. 2018; Kunarso *et al.* 2010; Schmid and Bucher 2010; Schmidt *et al.* 2012; Yue *et al.* 2014), and the binding of TFs to DNA is associated with an open and accessible chromatin state that can be identified with assays for transposase-accessible chromatin using sequencing (ATAC-seq) (Buenrostro *et al.* 2013, 2015). To determine whether TE-residing TF motifs in *C. elegans* are found within open chromatin sites, we compared the locations of TF motifs to previously published ATAC-seq data (Daugherty *et al.* 2017). In an effort to avoid any stage-specific biases that may be present, data from all available stages were considered (embryos, L3s, and young adults). Interestingly, we found that all TF motif types found within TEs occur within open chromatin sites at least once (Supplementary Table 4). To focus on accessible TF motifs with meaningful portions located within TEs, we restricted our further investigations to TF motifs with more than 10% of their accessible motifs are located within TEs (Fig. 3a). In order to prevent small numbers of motifs from yielding high percentages, we only considered TF motifs present in more than 3,000 open chromatin sites. From this analysis, we identified 22 different accessible motifs representing 19 TFs that occur within TEs in substantial numbers (Fig. 3a). Next, we examined the types of TEs that contribute the greatest numbers of accessible TF motifs and determined that a wide variety of TE types belonging to diverse TE families are responsible (Fig. 3b). Finally, we compared the positions of open-chromatin TF motifs to promoters, and found that 1,293 genes in total, or 6.5% of all protein-coding genes, have accessible motifs in TEs (Fig. 3c, Supplementary Table 5). Taken together, these results demonstrate that sizeable portions of TF-binding motifs within open chromatin are located within TEs found in the promoters of numerous protein-coding genes.

### Considerable portions of TF-binding sites are derived from TEs

**Fig. 4.**
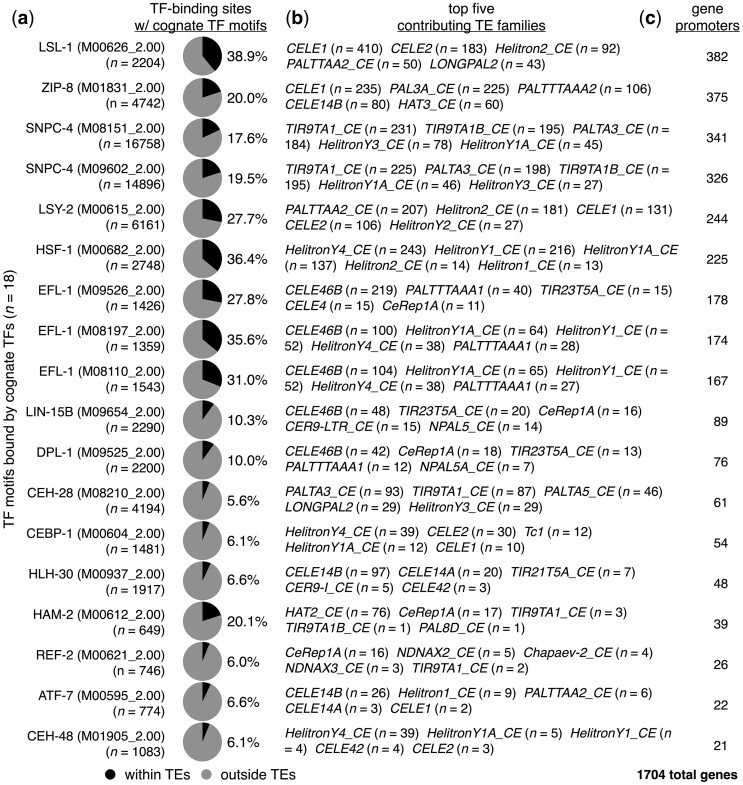
TF motifs within TEs are bound by their cognate TFs. a) Pie charts displaying the proportions of TF-binding sites with the indicated cognate TF-binding motifs that reside within (black) or outside of (gray) annotated TEs. TF-binding sites were identified using previously published ChIP-seq data (Li *et al.* 2016; Kudron *et al.* 2018). TF-binding motif identifiers from the Cis-BP database (Weirauch *et al.* 2014) are shown in parentheses. Only TF motifs with greater than 5% of TF-bound motifs within TEs are displayed. b) List of the top 5 TEs that have the greatest numbers of each indicated TF motif bound by their respective TFs. c) Number of protein-coding genes that have TEs containing TF-bound motifs from panel (a) residing within their promoter regions. The total number of genes with TE-provided TF-bound motifs in their promoters is 1,704.

Chromatin immunoprecipitation followed by high-throughput sequencing (ChIP-seq) allows for directly determining genomic regions bound by TFs of interest (Johnson *et al.* 2007). Previous work employed ChIP-seq to discover that noteworthy portions of TF binding sites in both human and mouse are derived from TEs (Sundaram *et al.* 2014). To determine whether TF-binding motifs within TEs are potentially bound by their cognate TFs in *C. elegans*, we determined the binding sites of 75 different TFs with corresponding Cis-BP motifs (Weirauch *et al.* 2014) using publicly available ChIP-seq data (Li *et al.* 2016; Kudron *et al.* 2018). In an effort to avoid any stage-specific biases that may be present, we combined binding sites obtained from all available stages for each TF. From this, we found that 86 different TF-binding motifs within 121 different types of TEs are bound by their cognate TFs (Supplementary Table 6). In addition, many of these TF-bound sites overlap with regions of accessible chromatin identified by ATAC-seq assays (*P<* 0.05, Fisher’s exact test) (Supplementary Table 7). To focus on TFs with notable portions of cognate binding events occurring within TEs, we focused our subsequent analyses to TFs with more than 5% of their binding sites containing TE-provided motifs (Fig. 4a). To prevent small numbers of TF sites from yielding high percentages, we only considered TFs with more than 500 bound regions. From this analysis, we identified 18 different motifs representing 15 TFs with more than 5% of their bound motifs within TEs (Fig. 4a). Next, we examined the types of TEs that contribute the greatest numbers of TF-bound motifs and determined that a wide variety of TE types are accountable (Fig. 4b). Finally, we compared the positions of TF-bound motifs within TEs to promoter regions, and found that 1,704 or 8.5% of all protein-coding genes have TE-derived TF binding sites (Fig. 4c, Supplementary Table 8). From this work, we conclude that substantial portions of binding sites for multiple types of TFs are derived from a diverse range of TEs that reside within promoter regions.

**Fig. 5.**
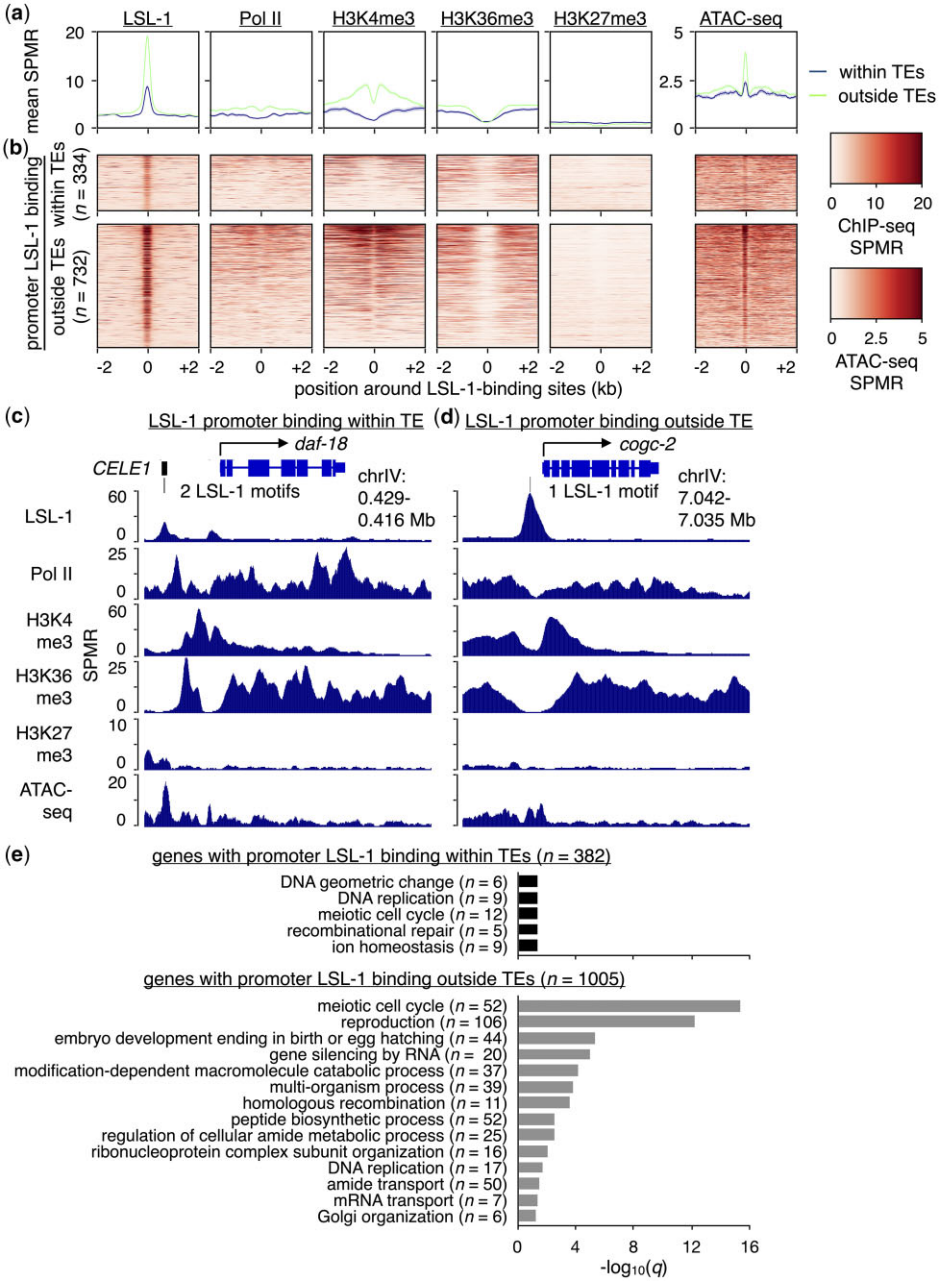
Genes with LSL-1-bound promoter TEs display a chromatin environment associated with gene expression. a) Plots showing the mean LSL-1, Pol II, H3K4me3, H3K36me3, and H3K27me3 ChIP-seq as well as ATAC-seq signal per million reads (SPMR) over LSL-1-bound regions containing LSL-1 motifs that reside either within (blue) or outside of TEs (green). LSL-1 ChIP-seq data were obtained from a previous publication (Kudron *et al.* 2018). Like LSL-1, histone modification and Pol II ChIP-seq data as well as ATAC-seq data were obtained from young-adult-staged worms (Contrino *et al.* 2012; Daugherty *et al.* 2017; Jänes *et al.* 2018). b) Heatmaps of LSL-1, Pol II, H3K4me3, H3K36me3, and H3K27me3 ChIP-seq as well as ATAC-seq SPMR over individual LSL-1-bound regions containing LSL-1 motifs. c) UCSC Genome Browser screenshot (Kent *et al.* 2002) with approximate genomic coordinates showing LSL-1, Pol II, H3K4me3, H3K36me3, and H3K27me3 ChIP-seq as well as ATAC-seq SPMR in the region surrounding the LSL-1-bound gene *daf-18*. Two *CELE1*-provided LSL-1-bound motifs are present within its promoter. d) UCSC Genome Browser screenshot with approximate genomic coordinates showing LSL-1, Pol II, H3K4me3, H3K36me3, and H3K27me3 ChIP-seq as well as ATAC-seq SPMR in the region surrounding the LSL-1-bound gene *cogc-2*. A single LSL-1-bound motif that resides outside of any annotated TEs is present within its promoter. e) Bar chart of significantly enriched GO terms and their corresponding −log_10_(*q*) values for genes with promoter LSL-1-bound regions containing LSL-1 motifs within (black) or outside of TEs (gray). Only GO terms associated with biological processes are shown.

The DNA-binding motif bound by its cognate TF and present in the most gene promoters was for LSL-1 (Fig. 4c), which has important roles in promoting embryonic viability (Fernandez *et al.* 2005). To determine whether genes bound by LSL-1 show evidence for active expression, we compared levels of published LSL-1 (Kudron *et al.* 2018) and young-adult-matched RNA polymerase II (Pol II) ChIP-seq data (Contrino *et al.* 2012), as well as histone modifications associated with gene expression (histone H3 trimethylated at Lysine 4 or H3K4me3, H3K36me3) or repression (H3K27me3) (Jänes *et al.* 2018) over regions surrounding 334 TE-derived and 732 non-TE-derived LSL-1 binding sites found in promoters (Fig. 5, a and b). In addition, we compared stage-matched ATAC-seq signal levels (Daugherty *et al.* 2017) over these regions to visualize chromatin accessibility (Fig. 5, a and b). Overall, promoters with TE-derived and non-TE-derived LSL-1 binding sites display hallmarks of active transcription, albeit to a lesser extent for the genes with TE-derived LSL-1 binding motifs. Individual examples of genes with LSL-1 binding to promoters within and outside of TEs are shown in Fig. 5, c and d. The promoter-TE-containing gene *daf-18* has 2 *CELE1*-provided LSL-1 motifs (Fig. 5c), while *cogc-2* has a single LSL-1 motif without any TEs present (Fig. 5d). These findings suggest that the binding of LSL-1 to promoter TEs is associated with active gene expression.

TEs can diversify transcriptional programs by bringing new genes with different functions under the control of a specific TF. To see if this may be the case with LSL-1-bound TEs, we compared enriched GO terms between genes with LSL-1 bound to TE and non-TE promoter regions. The 382 genes with LSL-1-bound promoter TEs (Fig. 4, Supplementary Table 8) are significantly enriched (*q* < 0.05) for 5 GO terms (“DNA geometric change,” “DNA replication,” “meiotic cell cycle,” “recombinational repair,” and “ion homeostasis”) after filtering for biological processes and redundancy using REViGO (Supek *et al.* 2011) (Fig. 5e). Only 31 genes contribute to any enriched GO terms (Supplementary Table 9), suggesting that few genes (8%) in this class have any common or known functionality. Conversely, the 1,005 genes with LSL-1 bound to non-TE promoter regions (Supplementary Table 10) are significantly enriched for 14 GO terms after filtering (Fig. 5e), with 393 genes (39%) contributing to any enriched terms (Supplementary Table 11). GO terms for functions such as “reproduction” and “embryo development ending in birth or egg hatching” are present (Fig. 5e), which is consistent with the embryonic lethality observed after depletion of LSL-1 (Fernandez *et al.* 2005). While genes with LSL-1 bound to TE- and non-TE-derived promoter regions largely display different GO terms, “DNA replication” and “meiotic cell cycle” are shared between these 2 classes, suggesting similar functionality for some genes (Fig. 5e). Of the 40 genes belonging to both classes, only 2 contribute to the “meiotic cell cycle” GO term. Taken together, our results suggest that genes with LSL-1 bound to promoter TEs are expressed and display differences in enriched GO terms, consistent with TE-mediated diversification of gene expression.

### Orthologous genes in *C. elegans* and *C. briggsae* have similar TF-motif-containing TEs in their promoter regions

**Fig. 6.**
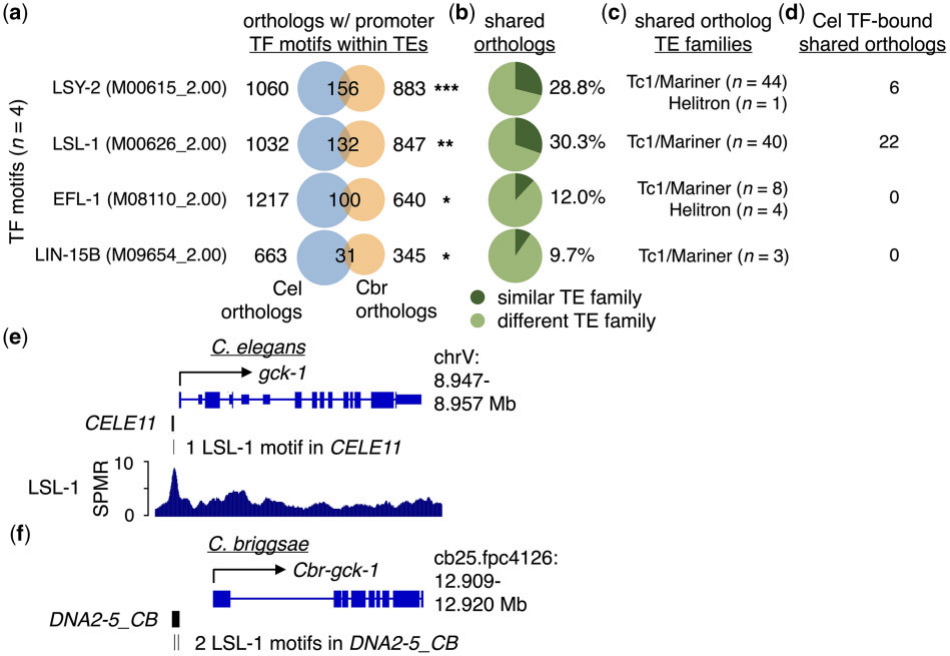
Orthologous genes in *C. elegans* and *C. briggsae* have TF-motif-containing promoter TEs. a) Venn diagrams depicting the number of one-to-one orthologs with promoter TEs containing the indicated TF motifs in *C. elegans* (Cel) (blue), *C. briggsae* (Cbr) (orange), or both species. Venn diagrams were generated in R using the VennDiagram v1.6.20 package (Chen 2018; R Core Team 2020). TF-binding motif identifiers from the Cis-BP database (Weirauch *et al.* 2014) are shown in parentheses. Only TF motifs with significant numbers of shared orthologs are shown. One asterisk represents *P <* 0.05, 2 asterisks represent *P <* 0.01, and 3 asterisks represent *P* < 1.0e−05 for the number of shared orthologs observed (hypergeometric test). b) Pie charts displaying the proportion of shared orthologs identified in panel (a) that have TF-motif-containing promoter TEs belonging to similar (dark green) or different TE families (light green). c) Identified TE families for TF-motif-containing promoter TEs that are similar between *C. elegans* and *C. briggsae* orthologs. d) Number of shared one-to-one orthologs with promoter TEs belonging to similar and different TE families bound by their cognate TFs in *C. elegans*, identified using previously published ChIP-seq data (Kudron *et al.* 2018). e) UCSC Genome Browser screenshot with approximate genomic coordinates of the region surrounding *gck-1* in *C. elegans*. The Tc1/mariner-related transposon *CELE11* containing 1 LSL-1-bound motif is found in its promoter. f) UCSC Genome Browser screenshot with approximate genomic coordinates of the region surrounding *Cbr-gck-1* in *C. briggsae.* The Tc1/mariner-related transposon *DNA2-5_CB* containing 2 LSL-1-bound motifs is found in its promoter.

Most TE insertions are deleterious or selectively neutral, with relatively few being beneficial and maintained over time (Sundaram and Wang 2018). To identify TE-derived TF-binding motifs that might be conserved between *C. elegans* and *C. briggsae* and, therefore, potentially functional, we searched for TE-derived motifs in promoter regions shared between these 2 species. To accomplish this, we scanned the *C. briggsae* genome (build cb4) for Cis-BP TF-binding motifs (Weirauch *et al.* 2014), as we had done with *C. elegans*, and identified all one-to-one orthologs shared between *C. elegans* and *C. briggsae* that contain TE-provided TF motifs in their putative promoter regions. We then determined whether the numbers of shared orthologs with TE-provided motifs were greater than expected by chance (*P<* 0.05, hypergeometric test). From this, we found motifs for 4 individual TFs (LSY-2, LSL-1, EFL-1, and LIN-15B) that are shared between orthologs in *C. elegans* and *C. briggsae* in significant numbers (Fig. 6a, Supplementary Table 12). Next, we determined the proportions of TF-motif-containing TEs in shared promoter regions that belong to similar TE families (Fig. 6b). Interestingly, we found that substantial portions of orthologous promoter TEs containing either LSL-1 or LSY-2 motifs belong to similar TE families (29% and 30%, respectively), and smaller portions of those with EFL-1 (14%) or LIN-15B motifs (12%) (Fig. 6b). After examining the TE families that are found in orthologous promoters between species, we found that the Tc1/mariner superfamily accounts for nearly all shared promoter TEs that were observed, while Helitrons account for the remaining TEs (Fig. 6c).

We then investigated if any TF-motif-containing TEs in shared promoter regions are bound by their cognate TFs in *C. elegans* using published ChIP-seq data (Li *et al.* 2016; Kudron *et al.* 2018). We found that 6 orthologs with shared promoter TEs containing LSY-2 motifs are bound by their cognate TF, while 22 orthologs with LSL-1 motifs are bound by their cognate TF (Fig. 6d); no orthologs were identified with EFL-1 or LIN-15B motifs bound by their corresponding TFs (Fig. 6d). An example of orthologous genes with shared promoter TEs containing LSL-1-binding motifs is depicted in Fig. 6, e and f. The *C. elegans* gene *gck-1* has the Tc1/mariner-related transposon *CELE11* with an LSL-1 motif bound by LSL-1 in its promoter region (Fig. 6e), while the *C. briggsae* ortholog *Cbr-gck-1* has 2 LSL-1 motifs within the Tc1/mariner-related transposon *DNA2-5_CB* (Fig. 6f). Taken together, our findings suggest that there are potential examples of promoter TEs with TF-bound motifs that have been conserved since the divergence of *C. elegans* and *C. briggsae*.

## Discussion

Here, we show that *C. elegans* TEs make up considerable portions of protein-coding gene promoters and harbor diverse TF DNA-binding motifs. Remarkably, numerous TE-derived TF motifs reside within accessible chromatin sites associated with TF binding and/or are bound by their cognate TFs, arguing for their functionality. In agreement with TE insertions promoting gene expression, genes with TE-derived LSL-1-binding sites in their promoters reside within a chromatin environment associated with gene expression similar to non-TE-derived sites. Furthermore, genes with TE-derived and non-TE-derived LSL-1 binding sites display differences in GO term enrichments. There are examples of orthologs shared between *C. elegans* and *C. briggsae* that contain similar TF-motif-containing TEs in their promoter regions, raising the possibility of conservation. As the first comprehensive analysis of TE-TF interactions in *C. elegans*, this work reveals that nematode TEs provide numerous TF-binding sites that have the potential for influencing gene expression.

### TEs harbor numerous and diverse TF DNA-binding motifs in *C. elegans*

The proliferation and accumulation of TEs in host genomes can provide a reservoir of diverse genetic material with the potential to be used for regulatory purposes. TEs make up substantial portions of most eukaryotic genomes (Wells and Feschotte 2020), with some specific examples being ∼85% in maize (Schnable *et al.* 2009), ∼44% in humans (Lander *et al.* 2001), and ∼20% in *Drosophila melanogaster* (Barrón *et al.* 2014). In *C. elegans*, TEs make up ∼9% of the genome, with 155 different types of annotated TEs (Kent *et al.* 2002; Smit *et al.* 2013). We found that all types of TEs have instances of TF-binding motifs, and of the 40,743 individual regions that have been identified as TEs or remnants thereof (Kent *et al.* 2002; Smit *et al.* 2013), 38,532 or roughly 95% contain TF-binding motifs. Of the 5.4 million total TF DNA-binding motifs that were identified in this study, approximately 10% reside within TEs. Thus, *C. elegans* TEs serve as a source of numerous and diverse TF-binding motifs. As TE-provided TF-binding motifs are found in the putative promoter regions of roughly 46% of all protein-coding genes, TE-derived regions have extensive potential for influencing the expression of nearby genes. Further investigation is required to determine whether individual TE-derived TF-binding motifs have roles in the regulation of gene expression.

Previously, we showed that a significant portion of HSF-1 TF binding motifs (known as Heat Shock Elements, or HSEs) reside within Helitron TEs (Garrigues *et al.* 2019). In this study, we found that in addition to HSF-1, 6 other TFs have greater than 10% of their genome-wide DNA-binding motifs within TE-derived regions (ZIP-3, ATF-7, ZIP-11, ATF-5, LSY-2, and LSL-1). We speculate that some TE-contributed TF-binding sites may have unconventional roles important for the regulation of gene expression, such as providing decoy sequences that regulate the amount of free unbound TF available for binding to target genes. Whether variability in the number of TEs with substantial amounts of TF-binding motifs affects the robustness of specific gene expression programs remains to be determined.

### TE-derived TF DNA-binding motifs display evidence for functionality

Since the groundbreaking work demonstrating that ∼30% of genome-wide human p53 binding sites reside within endogenous retroviruses (ERVs) and influence gene expression (Wang *et al.* 2007), numerous studies have identified widespread TE-derived TF-binding sites that influence gene expression networks in a variety of species. This study represents the first comprehensive genome-wide analysis of TF interactions with TE-derived regions in the important model organism *C. elegans*. We found TF-TE interactions to be extensive, with 15 individual TFs having greater than 5% of their binding sites within TE-derived regions. Of these, 7 TFs (LSL-1, ZIP-8, SNPC-4, LSY-2, HSF-1, EFL-1, and HAM-2) have at least 20% of their genome-wide binding sites in TE-derived regions, demonstrating that substantial numbers of TE-derived DNA-binding sites for multiple TFs are found within *C. elegans*. In addition, using regions of accessible chromatin as a proxy for TF binding, we identified an additional 13 TFs with greater than 10% of their accessible binding motifs residing within TE-derived regions. However, it remains possible that these open chromatin sites are due to the binding of unrelated factors or other mechanisms that affect chromatin accessibility. In most cases, TF-binding sites are not restricted to a single TE family but provided by multiple families, revealing that distinct TE families can simultaneously influence the genome-wide binding patterns of individual TFs. The TE-derived sites identified here are probably an underrepresentation of the total number present, as not all developmental stages were included in this analysis, and TE-TF interactions that occur in a limited number of cells or are transient in nature and may not be detectable in the data analyzed here. Whether these TE-provided TF-binding sites influence gene expression or are important for biological functions remains to be determined. This study provides the raw material for future work to determine the functional consequences of individual TE-derived binding sites for specific TFs of interest.

### Tc1/mariner TEs containing similar TF DNA-binding motifs are found in orthologous promoters

After comparing the genomes of *C. elegans* and *C. briggsae*, we found significant numbers of orthologous promoter pairs that contain TE-derived motifs for LSY-2, LSL-1, EFL-1, and LIN-15B. The presence of TE-derived TF-binding motifs in orthologous promoters suggests these genes have a propensity to acquire these motifs or possibly represent conservation. Most of these orthologous regions are derived from different TE families in each species and are therefore likely independent TE insertions rather than conservation, and may reflect “hotspots” where insertions are preferred or well tolerated. Whether these TE-derived motifs were already present within TEs during insertion or subsequently acquired through evolutionary forces is unknown. For orthologous TE-derived TF-binding motifs obtained from similar TE families, we found that the overwhelming majority are part of the Tc1/mariner superfamily, a diverse range of TEs found in a wide variety of species including animals, plants, and bacteria (Doak *et al.* 1994). Among the most active TEs in *C. elegans* (Eide and Anderson 1985; Plasterk *et al.* 1999; Laricchia *et al.* 2017), Tc1/mariner-related TEs are highly prevalent with 28 different families providing 7582 individual TE fragments that comprise almost 2% of the genome (Kent *et al.* 2002; Smit *et al.* 2013). Similarly, there are 80 different Tc1/mariner-related families in *C. briggsae*, which provide 17,643 individual elements that comprise roughly 4% of the genome (Harris *et al.* 2020). Due to the prevalence and diversity of Tc1/mariner-related TEs in both *C. elegans* and *C. briggsae*, it is unclear whether these orthologous promoter TEs with similar TF-binding motifs represent ancient insertions that have been conserved or independent insertions that occurred at different times.

While largely ignored in many genome-wide studies due to their repetitive nature, regions derived from TEs are major sources of TF-binding sites. Indeed, our prior work determined that roughly half of all protein-coding genes under the control of HSF-1 during heat stress are due to the presence of TE-derived binding sites (Garrigues *et al.* 2019). This work highlights the extensive potential that TEs have in influencing gene expression in *C. elegans* and provides a foundation for further investigation into the contributions of TEs to gene expression programs.

## Data availability

All data used in this study have been previously published and are publicly available. Accession numbers for all ATAC-seq data (Daugherty *et al.* 2017) and ChIP-seq data (Contrino *et al.* 2012; Li *et al.* 2016; Jänes *et al.* 2018; Kudron *et al.* 2018) used and their respective databases are listed in Supplementary Table 13.

Supplemental material is available at G3 online.

## Supplementary Material

jkac009_Table_S1

jkac009_Table_S2

jkac009_Table_S3

jkac009_Table_S4

jkac009_Table_S5

jkac009_Table_S6

jkac009_Table_S7

jkac009_Table_S8

jkac009_Table_S9

jkac009_Table_S10

jkac009_Table_S11

jkac009_Table_S12

jkac009_Table_S13

jkac009_Supplemental_Tables_Legends
