# Supplementary material for: Caenorhabditis elegans transposable elements harbor diverse transcription factor DNA-binding sites
Source: G3 (Bethesda). 2022 Jan 17;12(3):jkac009. doi: 10.1093/g3journal/jkac009 (PMC8896005; doi:10.1093/g3journal/jkac009)
Supplement: jkac009_Supplemental_Tables_Legends [file jkac009_supplemental_tables_legends.docx]

**SUPPLEMENTAL MATERIAL**

**Table S1. Occurrences of TF DNA-binding motifs in TE families.**

Table S1 includes the number, percent within, and fold enrichment of scanned TF motifs within TE families.

**Table S2. TF DNA-binding motifs in consensus TE sequences.**

Table S2 lists Cis-BP motif identifiers, their corresponding TFs, the number of times each TF motif is found in the consensus TE sequences, and TE family names.

**Table S3. Genes with TE-enriched TF DNA-binding motifs in their promoters.**

Table S3 lists the protein-coding genes from Figure 2 that have TE-derived TF-binding motifs in their putative promoter regions, and their corresponding Cis-BP motif identifiers and corresponding TFs.

**Table S4. Proportions of TF motifs within ATAC-seq summit regions that reside in TEs.**

Table S4 includes the percentages of each scanned TF motif within ATAC-seq summit regions that reside within TEs.

**Table S5. Genes with promoter TF DNA-binding motifs in ATAC-seq summits.**

Table S5 lists the protein-coding genes from Figure 3 that have TE-derived TF-binding motifs within ATAC-seq summit regions in their putative promoter regions, and their corresponding Cis-BP motif identifiers and corresponding TFs.

**Table S6. Proportions of TF motifs within cognate TF ChIP-seq summit regions that reside in TEs.**

Table S6 includes the percentages of each scanned TF motif within their cognate TF ChIP-seq summit regions that reside within TEs.

**Table S7. Overlapping TF ChIP-seq and ATAC-seq summit regions in TEs.**

Table S7 lists the Cis-BP motif identifiers, cognate TFs, number of ChIP-seq summits, number of ATAC-seq summits, number of overlapping summits, and the Fisher’s exact test *p*-value for the number of overlapping summits.

**Table S8. Genes with promoter TF DNA-binding motifs in cognate ChIP-seq summits.**

Table S8 lists the protein-coding genes from Figure 4 that have TE-derived TF-binding motifs within cognate ChIP-seq summit regions in their promoter regions, and their corresponding Cis-BP motif identifiers and cognate TFs.

**Table S9. Enriched GO terms for genes with TE-derived promoter LSL-1-binding sites.**

Table S9 lists the protein-coding genes from Figure 5 containing TE-derived promoter LSL-1-binding sites with significantly enriched GO terms.

**Table S10. Genes with LSL-1 bound to non-TE promoter LSL-1 motifs.**

Table S10 lists the protein-coding genes from Figure 5 that have non-TE-derived LSL-1 motifs bound by LSL-1 in their promoter regions.

**Table S11. Enriched GO terms for genes with non-TE-derived promoter LSL-1-binding sites.**

Table S11 lists the protein-coding genes from Figure 5 containing non-TE-derived promoter LSL-1-binding sites with significantly enriched GO terms.

**Table S12. Orthologous genes with similar promoter TE-derived TF DNA-binding motifs in *C. elegans* and *C. briggsae*.**

Table S12 lists the protein-coding orthologs from Figure 6 that have similar promoter TE-derived TF-binding motifs in their putative promoter regions, and their corresponding Cis-BP motif identifiers and corresponding TFs.

**Table S13. Accession numbers for all datasets used in this study.**

Table S13 lists the name, accession number, repository, and publication for all datasets used in this study.
